# The Role of Plasmacytoid Dendritic Cells in Innate and Adaptive Immune Responses against Alpha Herpes Virus Infections

**DOI:** 10.1155/2011/679271

**Published:** 2011-03-31

**Authors:** Philipp Schuster, Jan Bernardin Boscheinen, Karin Tennert, Barbara Schmidt

**Affiliations:** Institute of Clinical and Molecular Virology, German National Reference Centre for Retroviruses, Friedrich-Alexander-Universität Erlangen-Nürnberg, Schlossgarten 4, 91054 Erlangen, Germany

## Abstract

In 1999, two independent groups identified plasmacytoid dendritic cells (PDC) as major type I interferon- (IFN-) producing cells in the blood. Since then, evidence is accumulating that PDC are a multifunctional cell population effectively coordinating innate and adaptive immune responses. This paper focuses on the role of different immune cells and their interactions in the surveillance of alpha herpes virus infections, summarizes current knowledge on PDC surface receptors and their role in direct cell-cell contacts, and develops a risk factor model for the clinical implications of herpes simplex and varicella zoster virus reactivation. Data from studies involving knockout mice and cell-depletion experiments as well as human studies converge into a “spider web”, in which the direct and indirect crosstalk between many cell populations tightly controls acute, latent, and recurrent alpha herpes virus infections. Notably, cells involved in innate immune regulations appear to shape adaptive immune responses more extensively than previously thought.

## 1. Introduction

The human alpha herpes viruses comprise three different viruses: herpes simplex virus type 1 (HSV-1), type 2 (HSV-2), and varicella zoster virus (VZV) [1]. These highly cytopathic viruses are characterized by a short replication cycle, a broad cell tropism, and an efficient spread in cell culture. Importantly, they exhibit a distinct neurotropism, which, after primary infection at mucocutaneous sites, guides the viral particles along the peripheral sensory nerves to the dorsal root ganglia or the trigeminal ganglion, where they establish latent infection. Under circumstances of local or systemic immune suppression, alpha herpes viruses are reactivated and transported the same way, but in the reverse direction, to the epithelial surfaces. Primary HSV-1 and HSV-2 infections can manifest as stomatitis aphthosa, herpetic whitlow, and neonatal herpes acquired by passage through the maternal birth canal [2]. Reactivations are well known as cold sores, corneal, and genital herpes. Primary and recurrent VZV infections manifest as chickenpox and shingles, respectively [3]. In rare cases, alpha herpes viruses cause severe diseases such as encephalitis, acute retinal necrosis, and life-threatening systemic infections. It still remains a mystery why only a few individuals in the large cohort of seropositives are so severely affected by these viruses.

The first animal model to investigate the pathogenesis of acute, latent, and recurrent herpes viral infections was described by von Szily, who, after inoculation of herpes simplex virus into the anterior eye chamber of rabbits, observed the spread of inflammation along the optic nerve and infiltration of the uvea of the contralateral eye [4]. These results were confirmed by Whittum and colleagues in a mouse model [5], which paved the way for further experiments in which herpes virus pathogenesis was analyzed in knockout mice or by depletion of individual cell populations. 

The focus of this paper is the immune control of alpha herpes virus infections. In particular, we wanted to summarize the role of cells involved in innate and adaptive immune responses and to highlight their interactions in the efficient control of acute and recurrent herpes virus infections with respect to the current literature. The reader is also referred to excellent reviews by others which addressed similar or related aspects of alpha herpes virus infections [6–9]. The corresponding immune escape mechanisms of alpha herpes viruses were recently described by others [10].

## 2. The Spider Web—Immune Surveillance of Alpha Herpes Virus Infections

**Figure 1:**
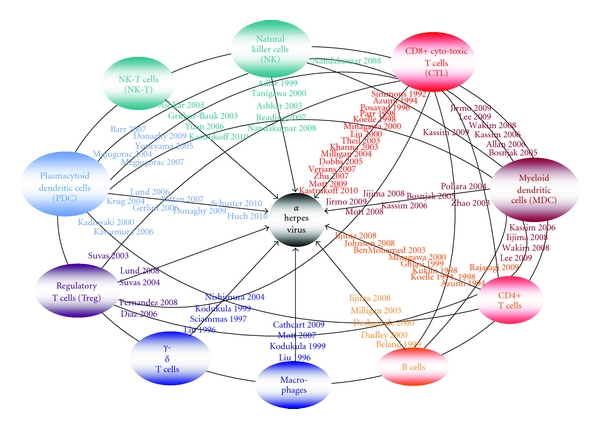
“Spider web” for the control of alpha herpes virus infections by cells of the innate and adaptive immune system (left and right side of the figure, resp.).

The current literature on the control of acute and latent herpes virus infections is summarized in Figure 1. There is not only evidence that single cell populations play a direct role in the suppression of alpha herpes virus replication, but cells interact with each other and across the innate-adaptive barrier in mediating efficient surveillance. 

### 2.1. Innate Immune Control of Acute Herpes Virus Infections

The resolution of acute herpes simplex infections is mostly associated with *macrophages and gamma delta T cells.* Liu and colleagues detected both cell types infiltrating the trigeminal ganglion after corneal HSV-1 infection in 1996 [11]. Mice depleted of gamma delta T cells or macrophages suffered from a more severe HSV-1 infection after footpad or ocular inoculation [12, 13]. Similar findings were obtained when the macrophage-derived inducible nitric oxide synthase was inhibited, and tumor necrosis factor- (TNF-) alpha or interferon- (IFN-) gamma secreted by macrophages and gamma delta T cells, respectively, were neutralized [12]. Mice deficient in gamma delta T cells were shown to be susceptible to intravaginal HSV-2 infection [14]. Depletion of macrophages in immunized mice increased the replication of HSV-1 in the eye of infected mice at early timepoints [15]. After injection of HSV-1 into the anterior eye chamber of BALB/c mice, an early influx of macrophages prevented the spread to the ipsilateral retina [16]. 

A further case was made for the role of *natural killer (NK) and NK-T cells* in the control of acute herpes simplex virus infections. Thus, it was shown that mice lacking NK and T cells suffered from more severe encephalitis after intranasal HSV-1 inoculation compared to mice only lacking T cells [17]. When HSV-1 was inoculated into the anterior eye chamber of NK-depleted BALB/c mice, the virus rapidly spread to the ispilateral retina [18]. Interleukin (IL-)18 was shown to be involved in the rapid activation of NK cells and thus in the control of early HSV-1 replication in the lung after intranasal infection of C57BL/6 mice [19]. NK-deficient mice also displayed a reduced quality and quantity of CD8+ cytotoxic T lymphocytes (CTL), which defines a novel helper role for NK cells [20]. C57BL/6 mice deficient in NK and NK-T cells were more susceptible to vaginal HSV-2 infection than control mice; these cells were the early source of IFN-gamma in the vaginal fluids [21]. In C57BL/6 mice lacking CD1d or NK-T cells, the clearance of HSV-1 inoculated by skin scarification was impaired compared to control mice [22]. These data are confirmed by a study showing that HSV-1 evades NK-T cell recognition by suppressing CD1d recycling [23]. The presence of NK-T cells, however, appeared to be less critical, when a less virulent strain of HSV-1 was used for infection [24].

### 2.2. Control of Herpes Virus Latency by Adaptive Immune Responses

In a study published in 1994, the inoculation of HSV-1 into the anterior eye chamber of BALB/c mice was followed by the infiltration of the ipsi- and contralateral retina after depletion of CD4+ and CD8+ T cells [25]. These data were extended by others, reporting that CD4+ or CD8+ T cell functions were required for the clearance of HSV-1 from trigeminal ganglia and establishment of latent nonlytic infection in mice [26]. In recurrent human genital HSV-2 lesions, HSV-specific T cell clones were recovered, the majority of which was positive for CD4 [27]. In a followup study, the authors showed that the viral clearance from the lesions was associated with a high level of local CD4+ and CD8+ T cell cytolytic activities towards HSV-infected cells [28]. 

Several reports focus on *CD4+ T cells* as the major cell population in the immune control of alpha herpes virus infections. In a study using T cell subtype knockout mice and mice depleted with T cell subset-specific antibodies, immunity against vaginal HSV-2 challenge after vaccination with recombinant vectors expressing viral glycoproteins was dependent on CD4+ T cells [29]. Transfer of CD4+ T cells (and to a minor extent CD8+ T cells) from immunized BALB/c mice into severe combined immunodeficient (SCID) mice contributed to latent HSV-1 infection in the trigeminal ganglia [30]. Another group identified immunodominant epitopes from the HSV-1 glycoprotein D that conferred Th1 CD4+ T cell protective immunity against lethal ocular challenge in a mouse model [31]. In CD8+ T cell-depleted and -deficient mice, CD4+ T cells gradually cleared infectious HSV-1 from neural tissues, not dependent on perforin or Fas [32]. This mechanism was confirmed in another study, in which effector CD4+ T cells protected mice from HSV-2 infection after recurrent vaginal challenge, using a noncytolytic mechanism via local IFN-gamma secretion [33].

Various studies emphasize the role of *CD8+ T cells* in controlling alpha herpes virus infections. In this respect, CD8+ T cells were shown to be responsible for the clearance of infectious HSV-1 virions from murine ganglia [34]. Mice depleted of CD4+ and CD8+ T cells, but also those depleted of only CD8+ T cells, suffered from a more severe HSV-2 infection in the vaginal epithelium than nondepleted immune mice [35]. A high frequency of HSV-specific CD8+ cytotoxic T cell precursors has been detected in patients suffering from genital herpes [36]. Addition of CD8alpha antibodies to *ex vivo* cultures of trigeminal ganglia caused HSV-1 reactivation from latency as evident from the expression of late proteins and infectious virions [37]. These data suggested ongoing viral replication at the sites of herpes virus latency, which was confirmed by detection of viral transcripts of the ICP4 and thymidine kinase genes in mouse ganglia [38, 39]. Others quantified the amount of virus replication and reported about 500 neurons with latency-associated transcripts in each trigeminal ganglion compared to one neuron with early and late viral transcripts and viral protein per 10 trigeminal ganglia [40]. Latent HSV-1 infection in human trigeminal ganglia was shown to be accompanied by a chronic inflammatory process [41], and CD8+ T cells specific for an immunodominant HSV-1 glycoprotein B epitope could block HSV-1 reactivation from latency in explanted trigeminal ganglion cultures [42]. The mechanism was shown not to be dependent on perforin- or Fas/FasL-mediated cytolytic pathways but on IFN-gamma secretion [43, 44]. Herpes virus latency in human trigeminal and genital ganglia was reported to be associated with local, persistent T cell responses against HSV-1 and HSV-2, respectively [45, 46]. The level of HSV-1 latency correlated with higher levels of PD-1 mRNA, suggesting an exhaustion of CD8+ T cells in the trigeminal ganglia of latently infected mice [47]. Notably, recent data show a diminished CD8+ T cell response in the draining lymph nodes of CD4+ T cell-depleted mice [48]. Altogether, CD4+ and CD8+ T cells appear to play a major role in maintaining alpha herpes virus latency by suppression of lytic replication. In neuronal tissues, this control is mostly effected through noncytotoxic immune responses.

Beland and colleagues showed in 1999 that *B cell* deficient mice succumbed to HSV-1 encephalitis compared to control C57BL/6 mice and that the transfer of hyperimmune sera protected from death [49]. SCID mice which were given immune serum prior to corneal HSV-1 infection survived without latent infection [30]. In B cell-deficient mice, vaginal HSV-2 infection and spread was limited by the transfer of immune sera [50], and the susceptibility to cutaneous HSV infection was increased up to 1000-fold in BALB/c and C57BL/6 mice with a defect in the *μ*-immunoglobulin chain [51]. In addition, virus-specific plasma cells were found over an extended period in sensory ganglia following intravaginal HSV-2 inoculation, which may contribute to latency [52].

### 2.3. Modulation of Adaptive Immune Responses by Regulatory T Cells (Treg)

The role of Treg in the control of alpha herpes virus infections is controversially debated. Publications focus on the suppressive role of Treg on CD4+ and CD8+ T cells [53, 54]. Thus, enhanced CD8+ T cell responses and a lower viral load were observed in mice depleted for Treg prior to HSV-1 inoculation into the foot pad [54]. In a followup study, a more severe immunoinflammatory stromal keratitis was induced after HSV-1 inoculation into Treg-depleted mice [55]. Another study reported enhanced CD8+ cell cytotoxicity and IFN-gamma responses by CD4+ and CD8+ T cells as well as reduced viral titers in the draining lymph nodes of Treg-depleted mice [56]. Into a different direction goes a recent report on Treg-depleted mice, which suffered from increased viral load and accelerated fatal infection after vaginal HSV-2 inoculation, suggesting that Treg coordinate early protective responses by allowing a timely entry of NK cells, DC, and T cells into the infected tissue [57]. The discovery of distinct surface markers of Treg, for example, GARP, may facilitate further studies about the role of these cells in controlling HSV infection [58].

### 2.4. Coordination of Innate and Adaptive Immunity by Dendritic Cells (DC)

There is ample evidence that myeloid and plasmacytoid DC directly and indirectly contribute to the control of alpha herpes virus infections. Mice which were inoculated intravaginally with HSV-2 showed a rapid recruitment of submucosal *myeloid dendritic cells (MDC)* to the infected epithelium, which, presenting viral peptides in the MHC class II context, emerged in the draining lymph nodes and stimulated IFN-gamma secretion from HSV-specific CD4+ T cells [59]. The binding of HSV-1 glycoprotein D caused type I IFN secretion and maturation of MDC [60]. Others reported that HSV-infected apoptotic DC were phagocytosed by uninfected bystander DC, which, after cross-presentation of viral antigens, stimulated virus-specific CD8+ T cells [61]. The depletion of CD11c+ DC in the highly resistant C57BL/6 strain resulted in enhanced susceptibility to HSV-1 infection and spread into the nervous system; the authors also observed an impaired activation of NK cells, CD4+ and CD8+ T cells [62]. The viral antigen appears to be taken up initially by migratory DC and then transferred to lymphoid-resident DC for presentation and CTL priming [63]. Immunization of mice with Flt3-ligand DNA increased the number of CD11c+ CD8alpha+ DC and enhanced HSV-1 latency [64]. In extra-lymphoid tissues, HSV-specific memory CD8+ T cell responses were stimulated through a tripartite interaction with CD4+ T cells and DC recruited from the blood [65]. Dependent on the mode of infection, lymph node-resident or tissue-derived migratory DC pick up and present HSV-1 epitopes to CD4+ and CD8+ T cells [66]. Recently, it was shown that the uptake of virus released from target cells led to cross-presentation of viral antigens, which was important for CTL immunity in mice [67]. The cooperation of dendritic cells and B cells restimulated memory CD4+ T cells to secrete IFN-gamma [33], and the depletion of DC impaired the magnitude of IFN-gamma expression and cytotoxicity of NK cells [68]. 

The importance of type I interferons has been highlighted in a study in which the expression of IFN-alpha in astrocytes protected mice from acute and latent ocular HSV-1 infection [69]. The type I IFN response of *plasmacytoid dendritic cells (PDC),* the major producers of type I IFN, to HSV-1 is mediated via the Toll-like receptor (TLR) 9 /MyD88 pathway [70]. However, mice lacking MyD88 or TLR9 control HSV-1 replication, indicating that TLR9-independent pathways are also involved [70, 71]. HSV-1 stimulation of PDC, concomitant to IFN-alpha production, stimulated naïve CD4+ T cells to produce IFN-gamma and IL-10 [72]. It also induced migration of activated T and NK cells by production of the chemokines CCL4 and CXCL10 [73]. In a mouse model of cutaneous HSV-1 infection, PDC-depleted mice showed a reduced CD8+ T cell IFN-gamma production and HSV-specific lysis accompanied by an increase in viral load; PDC alone failed to induce CTL, however provided efficient help to lymph node dendritic cells [74]. In mice vaginally inoculated with HSV-2, PDC were recruited to the site of infection; PDC-depleted and TLR9-deficient mice had survival curves which were significantly worse than wild type mice [75]. In the human system, HSV-stimulated PDC were shown to stimulate the differentiation of naïve CD4+ T cells into cytotoxic “regulatory” T cells, which expressed perforin and granzymes, and produced IFN-gamma and IL-10 [76]. Another group reported that PDC-derived IL-18 rapidly activated NK cells in HSV-1 infection [77]. Human PDC were recruited to varicella skin lesions [78, 79], and infiltrated the dermis of recurrent genital herpes simplex lesions, closely associated with NK and T cells [80]. HSV-infected monocyte-derived DC induced IFN-alpha production in PDC through the exchange of cellular material [81]. We have reported reduced PDC counts and function together with reduced CD8+ T cell IFN-gamma production in patients with HSV- and VZV-associated acute retinal necrosis [82]. However, it remained unclear whether these deficits were cause or consequence of uncontrolled virus replication. Altogether, evidence is accumulating that PDC are directly and indirectly involved in the control of acute and latent alpha herpes virus infections.

## 3. The PDC “Life Cycle”—A Reflection of the Surface Receptor Repertoire

What is the evidence that PDC—in addition to secretion of type I IFN and proinflammatory cytokines—shape the immune response by direct cell-cell contact? The antigen-presenting properties of PDC, which we are just beginning to understand, were recently addressed in an excellent review [83]. The PDC surface receptors are increasingly coming into the focus of scientific research [84–88]. However, data about the function of PDC surface receptors in alpha herpes virus infections are scarce, and in most cases the function is deduced from the regulation of expression upon stimulation of PDC with HSV, other viruses or CpG, and from the function of these surface receptors on other cell populations. 

**Figure 2:**
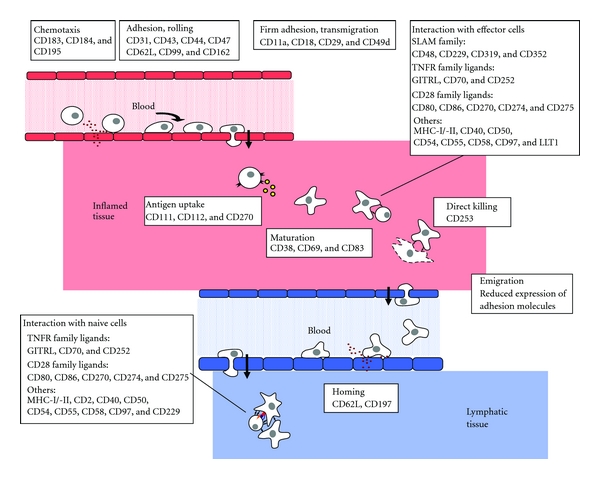
Proposed model for the plasmacytoid dendritic cell (PDC) “life cycle”, which is based on the timely coordinated expression of surface markers after contact with herpes simplex virus (HSV). The upper part of the figure depicts the attraction of PDC to inflamed tissue, where they take up their antigen and interact with local effector cells. Then, PDC reenter the blood stream and home to secondary lymphatic tissue via high endothelial venules (HEV), where they shape the adaptive immune response via interaction with naïve T cells, as outlined at the lower part of the figure.

Having these caveats in mind, we propose a model of the PDC “life cycle” (Figure 2), which was originally developed from chip and flow cytometry analyses of PDC in response to HSV-1 [87]. The timely coordinated regulation of PDC surface receptors suggests an inflammatory migration profile similar to gamma delta T cells [89], in which PDC are attracted to the site of infection, encounter their cognate antigen, reenter the blood stream, and then migrate to secondary lymphatic tissue. In more detail, immature PDC express three receptors for proinflammatory cytokines (CD183, CD184, and CD195), which were shown to be functional on these cells [90–92]. The presence of receptors involved in adhesion and rolling at the vascular endothelium (CD31, CD43, CD44, CD47, CD62L, CD99, and CD162) and those mediating firm adhesion and transmigration (CD11a, CD18, CD29, and CD49d) [93] suggest that PDC are recruited to inflamed tissue; explicit evidence is available for CD31 [92]. These data are corroborated by the detection of PDC in HSV and VZV lesions [75, 78–80]. At the site of infection, uptake of antigen may be mediated by three herpes virus entry mediators, namely CD111 (HVE-C), CD112 (HVE-B), and CD270 (HVE-A, HVEM) [80]. Other receptors relevant for antigen uptake may be CD4, the C-type lectin BDCA2 (CD303) [94, 95], Fc receptors [96–98], and CD170 (Siglec-5) [99]. Subsequent PDC activation and maturation can be seen from the enhanced expression of CD38, CD69, and CD83. Concomitant downregulation of adhesion molecules suggests that PDC can emigrate from the site of infection and reenter the blood stream. Homing to lymphatic tissue appears to be mediated by CD62L (L-selectin) [93, 100–102] and enhanced expression of the CC chemokine receptor 7 (CD197), which binds to CCL19 and CCL21 expressed by high endothelial venules in lymph nodes [90, 91, 103, 104]. Upon HSV-1 stimulation, CD197 was one of the few receptors which were upregulated with delay [87], suggesting that PDC indeed migrate from the site of infection to secondary lymphatic tissue. However, it has not been formally excluded that two different PDC populations migrate to different localizations.

The model of the PDC “life cycle” proposes two sites of interaction with other cells of the immune system: the inflamed tissue where PDC may encounter effector cells, and the lymphatic tissue, where PDC may interact with naïve cells and shape adaptive immune responses by the expression of coregulatory surface molecules. At the site of infection, expression of the SLAM family members CD48, CD229 (LY9), CD319 (CRACC), and CD352 (NTBA) may be particularly important for the interaction of PDC with NK and T cells [85, 87, 105–109]. CD319 may also induce proliferation of B cells [110]. This crosstalk appears to be mediated predominantly via homotypic receptor interactions [111]. 

Interaction of PDC with effector cells may also be influenced by the TNF receptor family [112]. PDC stimulated by virus or CpG express the ligand for the glucocorticoid-induced tumor necrosis factor receptor (GITRL), which may promote cytotoxicity and IFN-gamma production of CD357 (GITR)-expressing NK and T cells [113]. CpG-stimulated PDC fully activated NK-T cells involving direct contact of CD252 (OX40L) and CD134 (OX40) [114, 115]. CpG-activated PDC were also shown to promote B cell differentiation and immunoglobulin secretion via interaction of CD70 with CD27 [116].

A third family mediating coregulatory signals for naïve and memory T cells are the CD28 family ligands [112]. Of these, CD80, CD86, CD270, CD274 (B7-H1, PD-L1), and CD275 (B7-H2, ICOS-L) were described to be expressed on the PDC surface [80, 93, 95, 100, 117]. CpG- or HSV-stimulated PDC appear to drive CD4+ Treg immune responses via CD275 [117, 118], whereas CD40L-exposed PDC promoted the proliferation of CD8+ Treg [119]. 

CD50, CD54, and CD229 were reported to play an important role in the immunological synapse [120, 121], whereas the role of BDCA4 is still controversially discussed [122, 123]. PDC express the costimulatory molecules CD58 and CD97, which may costimulate NK and T cells [93, 124–126]. TLR9-activated PDC express the lectin-like transcript 1 (LLT1, CLEC2D), which interacts with CD161 on NK and effector T cells [127]. Recognition of virus-infected cells with subsequent induction of apoptotic and cytotoxic immune responses may be mediated by the enhanced expression of TRAIL (CD253) [128–130].

Several publications provide evidence for direct antigen presentation by PDC. After internalization and processing of influenza viruses, PDC cross-presented these antigens on MHC class I to CD8+ T cells [131]. Cross-priming of naïve CD8+ T cells with ovalbumin-exposed PDC was dependent on concomitant TLR activation [132]. Endocytosis of cytomegalovirus antigen together with CpG-B enhanced virus-specific memory CD4+ T cell responses [95]. In contrast, Yoneyama reported an indirect effect of PDC on the induction of CD8+ CTL by providing efficient help to lymph node DC via CD2 and CD40L in a model of cutaneous HSV-1 infection [74]. In addition, infiltrating CD8+ T cells may reciprocally influence PDC immune responses by secretion of IL-3 [133], which contributes to maturation and survival of PDC [100, 117].

## 4. The Risk Factor Model—Clinical Implications of Herpes Virus Reactivation

One of the best studied models of alpha herpes virus reactivation from latency is the acute retinal necrosis (ARN). Roughly one and two thirds of cases are caused by reactivation of HSV and VZV, respectively. Clinical course, treatment options and therapy outcome of this severe disease were recently reviewed [134–136]. The vasculitis, retinal necrosis, and intraocular inflammation start in the periphery and rapidly progress circumferentially, mostly affecting nonimmunocompromised patients at a frequency of 0.5 cases per 1 million. Current therapies combine antiviral and anti-inflammatory drugs with surgical intervention. An early vitrectomy with silicon oil instillation is associated with a lower incidence of retinal detachment compared to conservative treatment; however, the overall visual prognosis is poor [137, 138]. The cause appears to be, besides retinal detachment, herpes virus-associated retinal ischemia and atrophy of the optic nerve [139].

**Figure 3:**
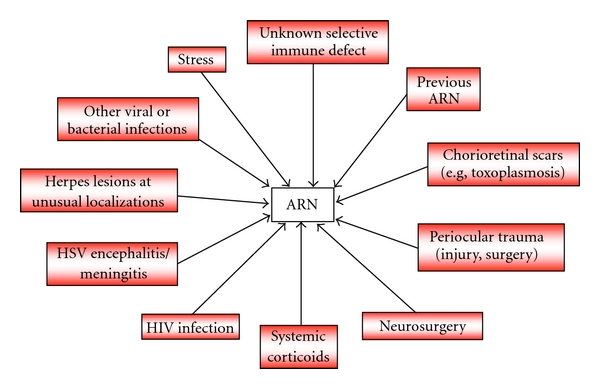
Risk factor model for the reactivation of latent herpes simplex (HSV) or varicella zoster virus infections leading to acute retinal necrosis (ARN).

In patients suffering from herpes virus-associated ARN, several risk factors can be identified (Figure 3). As such, there are previous ARN or chorioretinal scars from other diseases, for example, toxoplasmosis; periocular trauma; ocular; or brain surgery; previous herpes simplex virus encephalitis and iatrogenic or acquired immune deficiency caused by corticoid and/or antineoplastic treatment and HIV-1 infection, respectively [140–142]. In a systemic survey of nine patients with ARN, we additionally identified meningitis/encephalitis and frequent infections in childhood, recurrent herpes virus affections at unusual localizations in patients and relatives, as well as (bacterial) infections and stress around ARN [82]. These factors point to a local break in the integrity of ocular tissues and a weakened immune system distracted by other challenges. 

In addition, one may propose an (unknown) selective defect in the innate or adaptive immunity, which predisposes to alpha herpes virus reactivation. In mice, TLR9 plays a major role for the recognition of HSV-1 and HSV-2 although TLR-independent pathways have been described [70, 143]. Knockout mice for TLR2 and TLR9 were shown to be more susceptible to HSV-1 encephalitis [144, 145]. Similarly, the interferon response factor 3, which is in the downstream signaling cascade of endosomal and cytosolic recognition factors for double-stranded RNA, plays an important role in the control of HSV-1 replication [146, 147]. In humans, TLR9 is expressed on PDC and B cells, but—unlike the murine system—not on myeloid DC, which may explain why severe alpha herpes virus infections have not been associated with TLR9 polymorphisms in humans. Instead, defects in STAT1 [148], TLR3 [149], and the endoplasmatic reticulum transmembrane protein UNC93B have been reported in patients suffering from herpes simplex virus-associated encephalitis [150, 151].

## 5. Conclusions

What have we learned from the published data ? General conclusions are shortened in terms of the variability of animal models used: different animal strains, different viruses, and different modes of infection may lead to different conclusions. Another caveat is that mouse data do not readily translate into the human system, for which the data are still limited. Yet, three important conclusions can be drawn. First, many cell populations are involved to keep primary and recurrent alpha herpes virus infections under control, which may reflect how seriously these cytopathic infections are taken by the immune system. A second conclusion is that the textbook knowledge of innate cells controlling primary infection and adaptive immunity supervising latent alpha herpes virus infections is no longer clearcut. Cells involved in innate immune regulations appear to shape adaptive immune responses more extensively than previously thought. Is there a master regulator in this system, for example, the two dendritic cell populations that coordinate each other and all other cells ? Or does each component of the immune system substitute for another in some respect ? A third conclusion is that interactions of different cell populations via soluble factors and in particular via surface receptors appear to be crucial to fight against alpha herpes virus infections [152, 153], which, if not controlled efficiently, will cause severe disabling diseases.

## Abbreviations

ARN: Acute retinal necrosis
CCR: CC chemokine receptor
CTL: Cytotoxic T lymphocytes
DC: Dendritic cells
HSV: Herpes simplex virus
IFN: Interferon
IL: Interleukin
L: Ligand
MDC: Myeloid dendritic cells
NK: Natural killers
PDC: Plasmacytoid dendritic Cells
SCID: Severe combined immunodeficiency
TLR: Toll-like receptor
TNF: Tumor necrosis factor
Treg: Regulatory T cells
VZV: Varicella zoster virus.
